# Inferring causation from time series in Earth system sciences

**DOI:** 10.1038/s41467-019-10105-3

**Published:** 2019-06-14

**Authors:** Jakob Runge, Sebastian Bathiany, Erik Bollt, Gustau Camps-Valls, Dim Coumou, Ethan Deyle, Clark Glymour, Marlene Kretschmer, Miguel D. Mahecha, Jordi Muñoz-Marí, Egbert H. van Nes, Jonas Peters, Rick Quax, Markus Reichstein, Marten Scheffer, Bernhard Schölkopf, Peter Spirtes, George Sugihara, Jie Sun, Kun Zhang, Jakob Zscheischler

**Affiliations:** 1 German Aerospace Center, Institute of Data Science, Mälzer Str. 3, 07745 Jena, Germany; 2 Grantham Institute, Imperial College, London, SW7 2AZ UK; 3 Climate Service Center Germany (GERICS), Helmholtz-Zentrum Geesthacht, Fischertwiete 1, 20095 Hamburg, Germany; 4 Department of Environmental Sciences, Wageningen University, P.O. Box 47, NL-6700 AA Wageningen, The Netherlands; 5 Department of Mathematics, Clarkson Center for Complex Systems Science (C3S2), Clarkson University, 8 Clarkson Ave., Potsdam, NY 13699-5815 USA; 6 Image Processing Laboratory, Universitat de València, ES-46980 Paterna (València), Spain; 7 Department of Water and Climate Risk, Institute for Environmental Studies (IVM), VU University Amsterdam, De Boelelaan 1087, 1081 HV Amsterdam, The Netherlands; 8 Potsdam Institute for Climate Impact Research, Earth System Analysis, Telegraphenberg A62, 14473 Potsdam, Germany; 9 Scripps Institution of Oceanography, University of California, San Diego, 9500 Gilman Drive, La Jolla, CA 92093 USA; 10 Department of Philosophy, Carnegie Mellon University, 5000 Forbes Ave, Pittsburgh, PA 15213 USA; 11 Max Planck Institute for Biogeochemistry, PO Box 100164, 07701 Jena, Germany; 12 Department of Mathematical Sciences, University of Copenhagen, Universitetsparken 5, 2100 København, Denmark; 13 Institute for Informatics, University of Amsterdam, PO Box 94323, 1090 GH Amsterdam, The Netherlands; 14 Institute of Advanced Studies, University of Amsterdam, Oude Turfmarkt 147, 1012 GC Amsterdam, The Netherlands; 15 Max Planck Institute for Intelligent Systems, Max Planck Ring 4, 72076 Tübingen, Germany; 16 Department of Physics and Department of Computer Science, Clarkson University, 8 Clarkson Ave., Potsdam, NY 13699-5815 USA; 17 Institute for Atmospheric and Climate Science, ETH Zurich, Universitätstrasse 16, 8092 Zurich, Switzerland; 18 Climate and Environmental Physics, University of Bern, Sidlerstrasse 5, 3012 Bern, Switzerland; 19 Oeschger Centre for Climate Change Research, University of Bern, Bern, 3012 Switzerland

**Keywords:** Climate sciences, Environmental sciences, Computational science, Statistical physics, thermodynamics and nonlinear dynamics, Databases

## Abstract

The heart of the scientific enterprise is a rational effort to understand the causes behind the phenomena we observe. In large-scale complex dynamical systems such as the Earth system, real experiments are rarely feasible. However, a rapidly increasing amount of observational and simulated data opens up the use of novel data-driven causal methods beyond the commonly adopted correlation techniques. Here, we give an overview of causal inference frameworks and identify promising generic application cases common in Earth system sciences and beyond. We discuss challenges and initiate the benchmark platform causeme.net to close the gap between method users and developers.

## Abstract (web summary)

Questions of causality are ubiquitous in Earth system sciences and beyond, yet correlation techniques still prevail. This Perspective provides an overview of causal inference methods, identifies promising applications and methodological challenges, and initiates a causality benchmark platform.

## Introduction

Since Galileo Galilei, insight into the causes behind the phenomena we observe has come from two strands of modern science: observational discoveries and carefully designed experiments that intervene in the system of interest under well-controlled conditions. In one of Galilei’s early experiments—albeit a thought experiment^1^—, the law of falling bodies is discovered by dropping two cannonballs of different masses from the tower of Pisa and measuring the effect of mass on the rate of fall to the ground. Discovering physical laws this way is a challenging problem when studying large-scale complex dynamical systems such as the Earth system, because replicated interventional experiments are either infeasible or ethically problematic^2^. Surely, we should not conduct large-scale experiments on the Earth’s atmosphere: anthropogenic climate change already represents a rather uncontrolled long-term experiment. While randomized controlled experiments are a standard approach in medicine and the social sciences^3,4^, the main current alternative within most disciplines of Earth sciences are computer simulation experiments. However, these are very expensive, time-consuming, and require substantial amounts of expert knowledge, which in turn may impose strong mechanistic assumptions on the system^2^. Fortunately, recent decades have seen an explosion in the availability of large-scale time series data, both from observations (satellite remote sensing^5^, station-based, or field site measurements^6^), and from Earth system model outputs^2^. Such data repositories, together with increasing computational power^7^, open up novel ways to use data-driven methods for the alternative strand of modern science: observational causal discoveries.

In recent years, rapid progress has been made in computer science, physics, statistics, philosophy, and applied fields to infer and quantify potential causal dependencies from time series data without the need to intervene in systems. Although the truism that correlation does not imply causation holds, the key idea shared by several approaches follows Reichenbach’s common cause principle^8^: if variables are dependent then they are either causal to each other (in either direction) or driven by a common driver. To estimate causal relationships among variables, different methods take different, partially strong, assumptions. Granger^9^ addressed this question quantitatively using prediction, while in the last decades a number of complementary concepts emerged, from nonlinear dynamics^10,11^ based on attractor reconstruction, to computer science exploiting statistical independence relations in the data^4,12^. More recently, research in statistics and machine learning utilizes the framework of structural causal models (SCMs)^13^ for this purpose. Causal inference is growing to become a mature scientific approach^14^.

In contrast to data-driven machine learning methods such as probabilistic modeling^15^, kernel machines^16^, or in particular deep learning^17^, which mainly focus on prediction and classification, causal inference methods aim at discovering and quantifying the causal interdependencies of the underlying system. Although interpreting deep learning models is an active area of research^18^, extracting the causes of particular phenomena, e.g., hurricanes, from a deep learning black box is usually not possible. Therefore, causal inference methods are crucial in complementing predictive machine learning to improve our theoretical understanding of the underlying system^19^.

Unfortunately, many causal inference methods are still only known within a small community of methodological developers and rarely adopted in applied fields like Earth system sciences. Yet, data-based inference of causation was already proposed in the early 20th century by the geneticist Wright^20^, but it has not been widely adopted partly due to the fierce opposition of statisticians like Pearson^14^. In Earth system sciences, besides simulation experiments, (Pearson) correlation and regression methods are still the most commonly used tools. However, causal inference methods do have the potential to substantially advance the state-of-the-art—if the underlying assumptions and methodological challenges are taken into consideration.

With this Perspective, we aim to bridge the gap between potential users and developers of methods for causal inference. We discuss the potential of applying causal inference methods to four key generic problems that are also common in other fields: causal hypothesis testing, causal network analysis, exploratory causal driver detection, and causal evaluation of physical models. First, we provide examples where causal inference methods have already led to important insights in Earth system sciences before giving an overview of different methodological concepts. Next, we highlight key generic problems in Earth system sciences and outline new ways to tackle these within causal inference frameworks. These problems are translated into challenges from a methodological perspective. Finally, as a way forward, we give recommendations for further methodological research as well as new ways in which causal inference methods and traditional physical modeling can complement each other, in particular in the context of climate change research. This Perspective is accompanied by a website (causeme.net) hosting a causality benchmark platform to spur more focused methodological research and provide benchmarks useful not only in Earth system sciences, but also in related fields with similar methodological challenges.

## Example applications of causal inference methods

As in many other fields, methods based on correlation and univariate regression are still the most common data-based tools to analyze relationships in Earth system sciences. Such association approaches are useful in daily practice, but provide few insights into the causal mechanisms that underlie the dynamics of a system. Causal inference methods can overcome some of the key shortcomings of such approaches. In this section, we discuss application examples where causal inference methods have already led to important insights before providing a systematic overview.

**Fig. 1:**
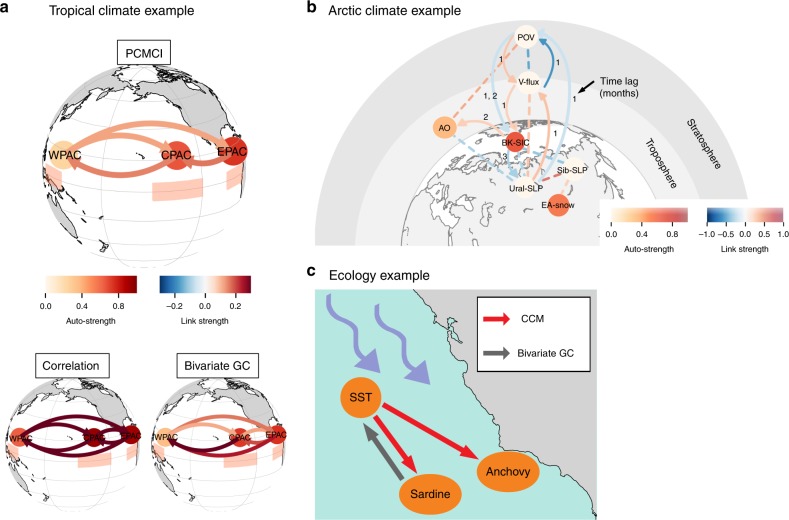
Example applications of causal inference methods in Earth system sciences. **a** Tropical climate example of dependencies between monthly surface pressure anomalies in the West Pacific (WPAC, regions depicted as shaded boxes below nodes), as well as surface air temperature anomalies in the Central Pacific (CPAC) and East Pacific (EPAC). Correlation analysis and standard bivariate Granger causality (GC) result in a completely connected graph while a multivariate causal method (PCMCI)^23,24^ better identifies the Walker circulation: Anomalous warm surface air in the East Pacific is carried westward by trade winds across the Central Pacific. Then the moist air rises towards the upper troposphere over the West Pacific and the circulation is closed by the cool and dry air sinking eastward across the entire tropical Pacific. PCMCI systematically identifies common drivers and indirect links among time-lagged variables, in this particular example based on partial correlation tests. Details on data in ref. ^53^. **b** Application of a similar method to Arctic climate^25^: Barents and Kara sea ice concentrations (BK-SIC) are detected to be important drivers of mid-latitude circulation, influencing winter Arctic Oscillation (AO) via tropospheric mechanisms and through processes involving vertical wave activity fluxes (v-flux) and the stratospheric Polar vortex (PoV). Details on methodology and data in ref. ^25^. ^©^American Meteorological Society. Used with permission. **c** Application from ecology (details in ref. ^11^): dependencies between sea surface temperatures (SST), and California landings of Pacific sardine (Sardinos sagax) and northern anchovy (Engraulis mordax). Granger causality analysis only detects a spurious link, while convergent cross mapping (CCM) shows that sardine and anchovy abundances are both affected by SSTs

Concurrently to Wright’s^20^ seminal works on causation in the 1920s, Walker was the first to introduce systematic correlation and regression analysis into climate science^21^. He discovered the temperature and pressure relationships between the East and West Pacific giving rise to the Walker circulation, which has by now been established not only from observational studies, but also detailed physical simulation experiments^22^. In Fig. 1a, we illustrate these relationships using different methods: classical correlation, standard bivariate Granger causality (GC), and PCMCI^23,24^ (described later) that is better suited to this problem. Whereas GC and standard correlation analysis results in unphysical links, the example demonstrates that with the correct application of an appropriate method the Walker circulation can be inferred from data alone.

Similary, Kretschmer et al.^25^ investigated possible Arctic mechanisms which could be pivotal to understand northern hemisphere mid-latitude extreme winters in Eurasia and North America. Arctic teleconnection patterns are much less understood than tropical ones and data-driven causality analyses are especially important because different climate models partly give conflicting results^26,27^. In Fig. 1b we highlight the Arctic teleconnection pathways of the stratospheric Polar vortex that were extracted from observational data alone: here causal inference methods have confirmed previous model simulation studies, finding that Arctic sea ice extent in autumn is an important driver of winter circulation in the mid-latitudes^28^.

Finally, Fig. 1c shows an example from ecology demonstrating that traditional regression analysis is unable to identify the complex nonlinear interactions among sardines, anchovy, and sea surface temperature in the California Current ecosystem. A nonlinear causal state-space reconstruction method^11^ here extracts the underlying ecologically plausible network of interactions, revealing that sea surface temperatures are a common driver of both sardine and anchovy abundances.

These examples demonstrate how causal inference methods can help in distinguishing direct from indirect links and common drivers from observational time series, while classical correlation methods are ambiguous to interpret and can lead to incorrect conclusions.

Next to Granger’s seminal works in economics^9,29^, observational causal inference methods have mostly been applied in neuroscience^30,31^ and bioinformatics^32,33^ where observational causal inference can also be combined with interventional experiments. The challenges for causal inference on Earth system data, especially the spatio-temporal and nonlinear nature of the system, are more similar to those in neuroscience as further discussed in the application and challenges sections.

## Overview of causal inference methods

Observational causal inference from time series has come a long way since Wiener’s^34^ and Granger’s^9^ seminal works in the 1950s and 1960s and a plethora of different methods have been developed since then. Importantly, in the past few decades the works of Pearl, Spirtes, Glymour, Scheines, and Rubin^3,4,12,35^ have grounded causal reasoning and inference as a rigorous mathematical framework, elucidating the conditions under which discovering causal graphical models, also called Bayesian networks^36^, from purely observational data is at all possible. These are known as identifiability conditions in the field of statistics and causal inference. Many causal inference methods for time series are grounded on the assumptions of time-order (causes precede effects), Causal Sufficiency, meaning that all direct common drivers are observed, and the Causal Markov Condition, stating that in a graphical model a variable *Y* is independent of every other variable (that is not affected by *Y*) conditional on *Y*’s direct causes, among other, more technical, assumptions^12,24^. However, recent work shows that some of these assumptions can be relaxed. Peters et al.^13^ summarize recent progress of methods that utilize assumptions on the noise structure and dependency types in the framework of SCMs. Many causal inference methods are not restricted to time series to infer causal relations.

### Granger causality

**Fig. 2:**
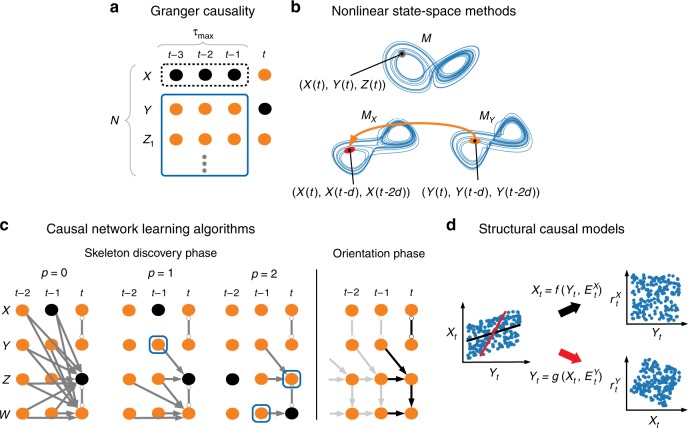
Overview of causal inference methods. **a** Multivariate Granger causality tests whether omitting the past of a time series *X* (black dashed box) in a time series model including *Y*’s own and other covariates’ past (blue solid box) increases the prediction error of *Y* at time *t* (black node). Hence, only time-lagged causal relations can be found. **b** The nonlinear state-space method convergent cross-mapping (CCM), illustrated for the chaotic Lorenz system, reconstructs the variables’ state spaces *(M*_*X*_*, M*_*Y*_*)* using time-lagged coordinate embedding and concludes on *X*→*Y* if points on *M*_*X*_ can be predicted using nearest neighbors in *M*_*Y*_ (orange ellipse) and the prediction improves the more points on the attractor are sampled. **c** Causal network learning algorithms cope well with high dimensionality and can often also identify the direction of contemporaneous links. Exemplified on the model of Box 1, the PC algorithm^12^, adapted to time series, starts from a graph where all unconditionally (*p* = 0) dependent variable pairs (assuming stationarity, only links ending at time *t* are represented) are connected and iteratively tests conditional independence with increasing number of conditions *p*. Lagged links are oriented forward in time (causes precede effects), while contemporaneous links are left undirected (circle marks at the ends) in this skeleton discovery phase. For example, *X*_*t*−1_ and *Z*_*t*_ (black nodes) are correctly identified as independent already in the second iteration step (*p* = 1) where the dependence through *Y*_*t-1*_ (blue box) is conditioned out, while we need to condition on two variables to detect that *Z*_*t*−2_ and *W*_*t*_ are independent (*p* = 2). In contrast to GC, PC avoids conditioning on the whole past leading to lower estimation dimensions. Contemporaneous links are then oriented by applying a set of rules in the orientation phase. Here the finding that *W*_*t-1*_ and *Z*_*t*_ are independent conditional on *Z*_*t*−1_, but not conditional on *W*_*t*_, allows to identify *Z*_*t*_→*W*_*t*_ because the other causal direction is not consistent with the observed conditional independencies. However, for the link between *X*_*t*_ and *Y*_*t*_ no such rule can be applied since all conditional-independence based algorithms resolve causal graphs only up to a Markov equivalence class. **d** Structural causal models utilize different assumptions than the previous approaches to detect causal directions within Markov equivalence classes by exploiting asymmetries between cause and effect (principle of independence of mechanisms^13^). Shown is the LiNGAM method^54^ (assuming a linear model with non-Gaussian noise) which can identify *Y*_*t*_$\rightarrow$*X*_*t*_ since the residual of the model for this direction (black fit line) is independent of *Y* (top subplot), while this is not the case for *X*_*t*_$\rightarrow$*Y*_*t*_ (red line)

The concept of Granger causality^9^ was the first formalization of a practically quantifiable causality definition from time series. The original idea, based on work by Wiener^34^, is to test whether omitting the past of a time series *X* in a time series model including *Y*’s own and other covariates’ past increases the prediction error of the next time step of *Y* (Fig. 2a). The concept of GC can be implemented with different time series models. Classically, the Granger causality test is based on linear autoregressive modeling (see Box 1), but nonlinear dependencies can be modeled with more complex time series models or even the information-theoretic analog transfer entropy^37^. While bivariate time series models do not explicitly account for indirect links or common drivers as shown in Fig. 1a, more variables can be included in multivariate extensions of GC. Nevertheless, as illustrated in Box 1 GC is limited to lagged causal dependencies and, furthermore, has known deficiencies in the presence of subsampled time series and other issues^38^. GC has a long history of applications across a wide range of scientific domains, including Earth system science^39–41^.

### Nonlinear state-space methods

While GC and also the other frameworks discussed here view systems as having interactions that arise from an underlying stochastic process, convergent cross-mapping^11^ (CCM) and related methods^10,42^ take a different dynamical systems perspective. These methods assume that interactions occur in an underlying dynamical system and attempt to uncover causal relationships based on Takens’ theorem and nonlinear state-space reconstruction. Thus, for these methods to apply it is necessary to demonstrate that a deterministic nonlinear attractor can be recovered from the data. In this sense it is thought to be complementary to the more statistical approaches discussed here. As illustrated in Fig. 2b, a causal relationship between two dynamical variables *X* and *Y* can be established if they belong to a common dynamical system, which can be reconstructed from time-delay embedding of each of the observed time series. More specifically, if variable *X* can be predicted using the reconstructed system based on the time-delay embedding of variable *Y*, then we know that *X* had a causal effect on *Y*. Nonlinear state-space methods have been applied to ecology^11,43^ as shown in Fig. 1c, as well as in climate science^44^.

### Causal network learning algorithms

For time series that are of a stochastic nature, CCM is less well suited. Multivariate extensions of GC fail if too many variables are considered or dependencies are contemporaneous due to time-sampling^24^ and in other cases (see also the challenges section). Causal network learning algorithms of various types have been developed for the reconstruction of large-scale causal graphical models. They can be classified by their search architecture, that is, whether they start with an empty or fully connected graph, and the statistical criterion for removing or adding an edge. The common feature of these algorithms is that they assume the Markov condition mentioned above together with the Faithfulness assumption, which requires that all observed conditional independencies arise from the causal structure^12^. Taken together, these two conditions allow to infer information about causal interactions from testing which conditional independencies hold true for the observed data. For example, the PC algorithm^45^ (named after its inventors Peter and Clark) and related approaches^23,24,46,47^ start with a fully connected graph and test for the removal of a link between two variables iteratively based on conditioning sets of growing cardinality (Fig. 2c). In this way also causal directions for contemporaneous links can often be assessed. Greedy equivalence search^48^, on the other hand, starts with an empty graph and iteratively adds edges. The statistical criterion for removing or adding an edge can either be a conditional independence test or a properly defined score function that quantifies the likelihood of a particular graph structure given the data. Conditional independencies can flexibly be tested with different types of tests: Linear conditional independence can be assessed with partial correlation, while a wealth of recent machine learning approaches on nonparametric tests addresses a wide range of independence and dependence types^24,49,50^. Score functions can be based on Bayesian or information-theoretic approaches. Sun et al.^51^, for example, cast causal network learning as an information-theoretic optimization problem. Causal network learning algorithms can incorporate time-order as a constraint (causes precede effects) and utilize a set of causal orientation rules to identify causal directions. The PC-based method PCMCI^23,24^ applied in Fig. 1a addresses the particular challenges of autocorrelated high-dimensional and nonlinear time series data based on a condition-selection step (PC), followed by the momentary conditional independence (MCI) test. As illustrated in Box 1, some network learning approaches, e.g., FCI^12^, account for unobserved direct common drivers and can still partially identify which links must be causal. Causal network learning algorithms have started to be applied in Earth system sciences only recently, mainly focusing on climate science^23,25,52,53^.

### Structural causal model framework

GC requires a time delay between cause and effect to identify causal directionality. If causation occurs almost instantaneously, or at least faster than the observable sampling interval, then causal directions cannot be identified in general. Many causal network learning algorithms, on the other hand, are also applicable to contemporaneous dependencies, but they can only identify causal graphs up to a Markov-equivalence class. For example, under the Faithfulness assumption, measuring that *X* is conditionally independent of *Y* given *Z*, while all other (conditional) relationships are dependent, gives rise to three different causal graphs that are Markov-equivalent if no additional information about time-order is available: *X*$\leftarrow$*Z*→*Y, X*→*Z*→*Y*, or *X*$\leftarrow$*Z*$\leftarrow$*Y*. As illustrated in Box 1, the simplest example of Markov equivalence are two contemporaneously dependent variables where the causal direction cannot be inferred with conditional independence-based methods. Structural causal models (SCMs) (Fig. 2d) can identify causal directions in such cases because they permit assumptions about the functional class of models (e.g., linear or nonlinear, additivity, noise distributions)^54–56^. Other methods exploit heterogeneity in the data by searching for models that are invariant over space or time^57–61^. For an overview see references^13,38^. Most of these principles extend to settings with temporal dependence as further elaborated in the Way forward section. SCMs have not yet been applied in Earth system sciences except for one work in remote sensing^62^.

Box 1 very short introduction to causal inference

Consider the time-dependent causal relations

1 $$X_t =	 aY_t + E_t^X\\ Y_t =	 E_t^Y\\ Z_t =	 bZ_{t - {\it{1}}} + cY_{t - {\it{1}}} + E_t^Z\\ W_t =	 dW_{t - {\it{1}}} + eZ_t + E_t^W,$$

with nonzero coefficients and where the noise terms *E*_*t*_^*Z*^, *E*_*t*_^*W*^ are standard normal and *E*_*t*_^*X*^, *E*_*t*_^*Y*^ uniformly distributed. The causal relations of this model are visualized in a time series graph (see Figure, panel a) with the repeated grey links indicating stationarity. The model features autocorrelation, lagged, and contemporaneous links that can emerge due to time aggregation (Fig. 4).

Lagged correlation (see Figure, panel b) here yields spurious associations between *X* and *Z* due to *Y* acting as a common driver. Furthermore, *Y* and *W* are correlated via an indirect path *Y*$\rightarrow$*Z*$\rightarrow$*W*, and *X* and *W* are also spuriously correlated. Multivariate Granger causality is designed to account for common drivers and indirect links and can be implemented as a vector autoregressive model. In the present example (see Figure, panel c), *Y* Granger-causing *W* is concluded by evaluating the two models

2 $$W_{t} = {\mathop{\sum}\limits_{\tau = 1}^{\tau_{\mathrm{max}}}} {\boldsymbol{\beta}}_{\tau} {{\bf{V}}}_{t - \tau } + \alpha_{\tau} Y_{t - \tau } + {\rm{error}}_{t}$$

3 $$W_t = {\mathop{\sum}\limits_{\tau = 1}^{\tau_{\mathrm{max}}}} { \tilde{\boldsymbol{\beta}}}_{\tau} {\bf{V}}_{t - \tau } + {\rm{error}}_{t}$$

with **V** = *(W*, *Z*, *X)* and establishing that the residual variance of model (2) is smaller than that of model (3). Put more generally, the information in the past of *Y* helps in predicting *W* beyond the remaining past (Fig. 2a). However, the link *Y*$\rightarrow$*W* is spurious since *Y*_*t*−1_ improves predicting *W*_*t*_ only indirectly via the contemporaneous *Z*_*t*_ and GC does not account for contemporaneous confounders or mediating variables. Furthermore, GC misses the contemporaneous causal relation *Y*_*t*_$\rightarrow$*X*_*t*_ because only information from the past is tested.

The PC algorithm represents the framework of causal network learning algorithms^12^ and overcomes some of these shortcomings. As explained in Fig. 2c the PC algorithm, adapted to time series, detects common drivers and indirect links also between contemporaneous variables. It unveils all spurious links and identifies the links *Y*_*t*−1_$\rightarrow$*Z*_*t*_ and *Z*_*t*_$\rightarrow$*W*_*t*_ (see Figure, panel d), while the link between *X*_*t*_ and *Y*_*t*_ cannot be oriented since the PC algorithm, and conditional independence-based network learning algorithms in general, can only detect causal graphs up to their Markov equivalence class (marked by circles at the end of links).

SCMs allow to identify causal directions also within a Markov equivalence class, if certain assumptions on the structural form of the underlying process are fulfilled. Shown here (see Figure, panel e) is the LiNGAM approach (explained in Fig. 2d) which can be adapted to time series and assumes that the model is linear and at least one of the noise terms is non-Gaussian. Here, LiNGAM can identify the linear causal influence *Y*_*t*_$\rightarrow$*X*_*t*_ because *X* and *Y* are driven by non-Gaussian noise. Note that already the presence of autocorrelation in either *X* or *Y* would allow the PC algorithm (but not GC) to identify the causal direction between the two. CCM, as a method that does not explicitly condition on other variables, is not well suited for multivariate, purely stochastic processes^24^.

The preceding analysis was based on methods whose output can be interpreted in a causal sense only under the assumption of Causal Sufficiency, that is, that no unobserved common drivers exist. The Fast Causal Inference (FCI) algorithm^12,47^ belongs to the class of network learning algorithms that do not require Causal Sufficiency. Like the PC algorithm, FCI is based on iterative conditional independence tests followed by (more involved) additional phases. Suppose FCI outputs the causal graph shown in the Figure in panel f. Here, the link between *X*_*t*_ and *Y*_*t*_ still cannot be oriented, and also for the link *Y*_*t−*1_$\rightarrow$*Z*_*t*_ we cannot exclude the possibility that a common driver induced this link (as marked by the circle at the tail of the link which stands for the two possibilities $\rightarrow$ and $\leftrightarrow$, the latter denoting a common driver link). However, the FCI output *Z*_*t*_$\rightarrow$*W*_*t*_ (without a circle at the tail) tells us that *Z*_*t*_ causes *W*_*t*_, potentially indirectly, but there cannot be a common driver since such a confounder would induce dependencies that are not consistent with the observation that here *Y*_*t*−1_ is conditionally independent of *W*_*t*_ given *Z*_*t*_ (or also that *Z*_*t*−1_ is conditionally independent of *W*_*t*_ given *Z*_*t*_ and *W*_*t*−1_).

This example demonstrates that even for very general cases, and without assuming away unobserved drivers, causal inference methods can extract causal information from observed conditional independencies and potentially further model assumptions. In practice, however, for short sample sizes some methods may strongly suffer from unreliable graph estimates.

## Key generic problems in Earth system sciences

### Causal hypothesis testing

**Fig. 3:**
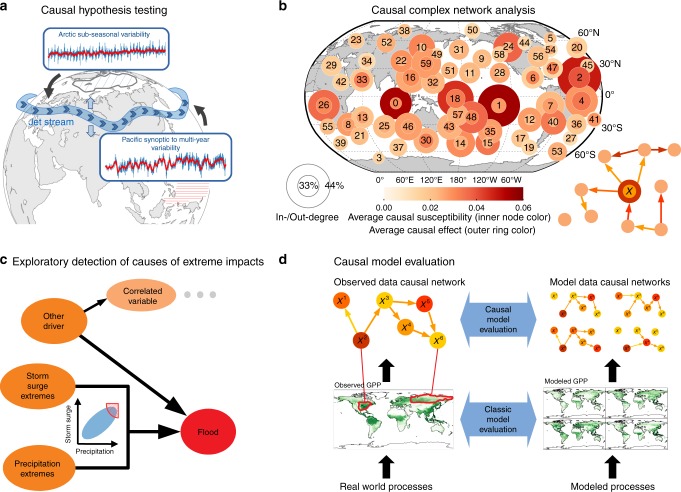
Key generic problems in Earth system sciences. **a** Causal hypothesis testing in climate research. The question of how the position of the jet stream depends on Arctic and tropical drivers is challenging due to different temporal scales and the spatial definition of variables (hatched regions). **b** Climate network analysis attempts to describe dynamics of the Earth system using complex network theory. Basing this theory on causal network measures allows one to better interpret network properties. Here major tropical atmospheric uplifts were identified as causal gateways with strong average causal effect and average causal susceptibility in the network (more details in ref. ^66^). Nodes correspond to climatic subprocesses in different regions and the lower right graph illustrates the causal network metrics for a variable *X*: the average causal effect is the average change in any other component (node) induced by a one-standard-deviation increase (perturbation) in *X*. Conversely, the average causal susceptibility is the average change in *X* induced by perturbations in any other component. Here, the Out-Degree refers to the fraction of components significantly (at 5% level) affected by a component and correspondingly for the In-Degree. **c** Identifying drivers of extreme impacts is challenging due to the typically large amount of correlated drivers compared to much fewer causally relevant drivers, that, furthermore, may only in combination have a large effect (synergy). For example, a flood might require both storm surges and precipitation to be in an extreme state. Such types of dependencies are difficult to represent with a pairwise network. **d** Basing model evaluation on causal statistics allows to better identify models with similar causal interaction structure as observational data, rather than comparing averages and climatologies. Shown is gross primary production (GPP) from observations and four illustrative models where the challenge lies in the extraction of variables *(X*^1^, *X*^2^, …), here shown by some red encircled regions, as well as defining suitable network comparison metrics (panel b) based on causal link weights (edge colors) and aggregate node measures (node colors)

We start by illustrating the challenges associated with a key causal hypothesis testing problem in climate research. Mid-latitude weather (including extreme events) is largely determined by nonlinear dynamical interactions between jet streams, storm tracks, and low-frequency teleconnections^63^. These dynamical processes are partially not well represented in the latest climate models. Hence, understanding drivers and favorable boundary conditions of weather-determining circulation regimes is crucial to improve (sub-)seasonal predictions, evaluate climate models, and reduce uncertainty in regional climate projections^64^. Important questions (Fig. 3a) in this context include: what drives the strength, position, and shape of the jet stream? What is the relative importance of tropical and Arctic processes^26,28,65^? Uncovering causal relations from the observational record here raises a number of challenges. To name just a few, first, time series representing the climatologically relevant subprocesses need to be extracted from typically gridded spatio-temporal datasets^25,66^, as illustrated in Fig. 3a. This can, for example, be achieved by averaging over corresponding regions, defining an index describing the jet stream position, or a more data-driven approach using dimension-reduction methods^66^. Secondly, reconstructing the causal relations between these extracted variables is challenging because different nonlinear processes can interact on vastly different time scales from fast synoptic and cloud-radiative processes to multi-year variability driven by slow oceanic processes^67^. Last, the distributions of climate variables, for example precipitation, are often non-Gaussian. Similar data characteristics also occur in neuroscience where first different subprocesses of the brain need to be reconstructed, e.g., from spatio-temporal electroencephalography measurements, and time series reflect a multitude of processes operating on different frequencies^30,68^.

### Causal complex network analysis

Network analysis of complex systems is a rapidly growing field^69^ and the network perspective may help to identify aggregate and emergent properties of the human brain^68^ or the Earth system^66^. For example, a phenomenon such as El Niño results from the complex interplay between multiple processes in the tropical Pacific^70^ and has a large effect on the global climate system. In standard approaches^68,71^, nodes are defined as the time series at different grid locations and links are typically based on correlations between the grid point time series. A common network measure is the node degree, which quantifies the number of processes linked to a node. However, defined based on correlations, network measures^69^ do not allow for a causal interpretation such as the information flow within the system^71^. Grounding network theory in causal networks allows to better interpret network measures^66,72^: an example for linear measures is reproduced in Fig. 3b. Like for the other generic problems, the challenges lie in high-dimensional nonlinear spatio-temporal data, and here also in a proper definition of network measures that takes into account causal interactions and accounts for the spatial definition of nodes. Causal network comparison metrics can then be utilized for a causal evaluation of physical models (see last paragraph in this section).

### Exploratory detection of causes of extreme impacts

In the Earth system, as well as in many other complex systems, the most devastating impacts are often related to multiple, compound or synergistic drivers^73^. For instance, devastating wildfires need dry and hot conditions, available fuel, and an ignition source. Many impacts are related to threshold behavior^74^, and multiple drivers contribute to the tipping of the system^75,76^. Consider the example shown in Fig. 3c where only the synergistic combination of extreme inland precipitation and extreme storm surge leads to coastal floods^77^. Causal inference methods can be helpful in identifying the relevant drivers from a typically large number of potential drivers that may be correlated with impacts^78^. Causal methods further allow us to identify regime shifts in functional relationships that are, e.g., triggered by extreme conditions. The challenges here include high-dimensionality, synergistic effects, and the often small sample size of observed impacts, and are relevant also in other fields such as neuroscience^68^.

### Causal evaluation of physical models

In many disciplines of Earth system sciences, models of the system or subsystem play a fundamental role in understanding relevant processes. Models differ regarding which subprocesses are resolved and the type of parametrization used. Biogeochemical models, for instance, help to understand element cycles and are a crucial basis for carbon-climate feedbacks in the coupled Earth system. At a higher level, climate models^2,79^ simulate the interactions of the atmosphere, water bodies, land surface and the cryosphere. In all cases, and at all levels, models are based partly on differential equations representing known processes and partly on semi-empirical relationships representing unknown processes or approximating known processes that cannot be resolved at the global scale due to numerical issues^80^. Due to the nonlinear nature of the system, small differences in parameterization can potentially lead to large deviations in overall model characteristics. A key task is to evaluate which model better simulates the real system. Currently, such evaluations are based on simple descriptive statistics like mean and variance, climatologies, and spectral properties of model output and observations^2,79^. However, even though a particular model might well fit descriptive statistics of the observational data, for example, the global distribution of gross primary production (GPP) (Fig. 3d), the model might not well simulate the physical mechanisms affecting GPP, given that multiple model formulations and parameterizations, even when wrong, can fit the observations equally well, a problem known as underdetermination or equifinality^81^. As a complementary criterion we propose to compare reconstructed causal dependencies of models and observational data (Fig. 3d). The underlying premise is that causal dependencies are more directly linked to the physical processes and are, therefore, more robust against overfitting than simple statistics and, hence, models that are causally similar to observations will also yield more reliable future projections. As for the previous example, also here the challenges lie in extracting suitable causal variables from often noisy station-based measurements or high-dimensional spatio-temporal fields and also the fact that processes can interact nonlinearly involving different spatio-temporal scales. In addition, model output may not satisfy the conditions underlying some causal inference methods, e.g., if dependencies are purely deterministic. Finally, suitable evaluation and comparison statistics based on causal networks need to be defined (see paragraph on causal complex network analysis). In Earth system sciences, model evaluation can help to build more realistic models to improve projections of the future, which is highly relevant for policy making^82^.

## Challenges from a methodological perspective

### Process challenges

**Fig. 4:**
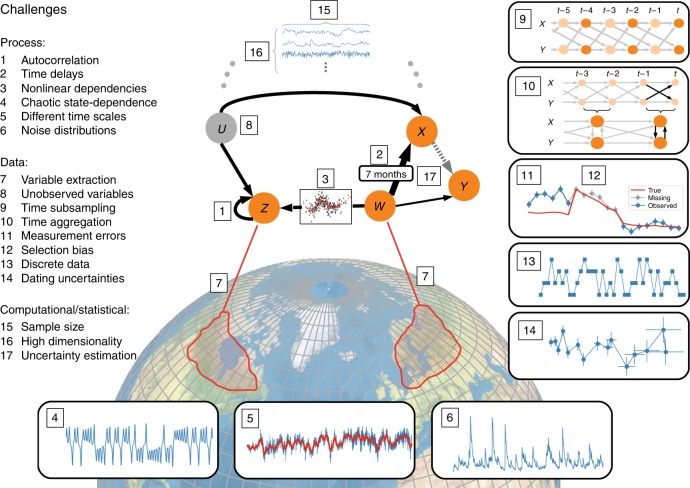
Methodological challenges for causal discovery in complex spatio-temporal systems such as the Earth system. At the process level, autocorrelation (1), time delays (2), and nonlinearity (3), also in the form of state-dependence and synergistic behavior (4), require a careful selection of the estimation method. Further, a time series might contain signals from different processes acting on vastly different time scales (5). Noise distributions (6) can feature heavy tails and extreme-values which challenges the ubiquitous methodological Gaussian assumption. At the data aggregation level, the most basic challenge is the definition of the causally relevant variables (7) representing the subprocesses of interest from spatio-temporally gridded data (e.g., from satellites) or station data measurements. Unobserved variables (8) need to be taken into account regarding a causal interpretation of the estimated graph. Time sub-sampling (9) and aggregation (10) can make causal links appear contemporaneous and even cyclic due to insufficient time resolution (e.g., due to the standard practice of time averaging depicted here in a time series graph^24^). Causal inferences are degraded due to measurement errors (11) such as observational noise, systematic biases (first few samples), or even missing values (grey samples), that may be causally related to the measured process, constituting a form of selection bias (12). Some datasets are of a discrete type (13), either due to quantization, or as categorical data, e.g., an index representing different weather regimes, and require methods that deal with discrete, and also mixed data types. Next to measurement value uncertainties, for paleo-climatic data even the measurement time points typically are given only with uncertainty (14), which especially challenges methods exploiting time-order. At the computational and statistical level, the scalability of methods, regarding both sample size (15) and high dimensionality (16) due to the number of variables as well as large time delays, is of crucial practical relevance for computational run-time and detection power. Finally, uncertainty estimation (17, width of links), also taking into account data uncertainties, poses a major challenge

At the process level, a number of challenges arise due to the time-dependent nature of the processes giving rise to strong autocorrelation (Fig. 4, point 1) and time delays (Fig. 4, point 2). Next, ubiquitous nonlinearity (Fig. 4, point 3), also in the form of state-dependence (Fig. 4, point 4) and synergy (see Fig. 3c), requires a careful selection of the estimation method (see nonlinear methods in method overview section). Note that sometimes variables from model output can be deterministically related via a set of equations, which poses a serious problem for many, but not all, causal methods^12,24^. As mentioned in the jet stream example, a geoscientific time series will typically contain signals from different processes acting on vastly different time scales, e.g., oceanic and atmospheric ones, which may need to be disentangled to better interpret causal links (Fig. 4, point 5). A basic assumption in a number of statistical methods used in causal inference frameworks (e.g., linear regression) is the assumption that the noise distribution is Gaussian, which is violated by processes featuring heavy tails and extreme events (e.g., precipitation; Fig. 4, point 6). On the other hand, some methods turn non-Gaussianity into an advantage^54^ (Fig. 2d).

### Data challenges

At the data aggregation level, our generic examples demonstrate that a major challenge is to define and reconstruct the causally relevant variables that represent the subprocesses of interest (Fig. 4, point 7). These variables have to be extracted from typically high-dimensional spatio-temporal gridded datasets (e.g., from satellite observations or model output) or station data measurements, which can be done by dimensionality reduction methods. Moreover, these extracted variables should be interpretable and represent physical subprocesses of the system.

Often, relevant drivers cannot be measured, which requires to consider the possibility of unobserved variables (Fig. 4, point 8) regarding a causal interpretation of the estimated graph, since they may render detected links spurious (see also Box 1). Arguably, identifying the absence of a causal link, implying that a physical mechanism is unlikely^24^, is a more robust finding, which requires less strong assumptions (no Causal Sufficiency). Another aspect of Causal Sufficiency is that not taking into account important drivers, such as anthropogenic climate forcings, may render time series nonstationary. Time series pose a particular challenge regarding time-subsampling (Fig. 4, point 9), which can also be considered as a case of unobserved samples of a variable, and time-aggregation (Fig. 4, point 10) which can let causal dependencies appear contemporaneous or even cyclic. The standard GC cannot deal with contemporaneous links, which can be identified using network learning algorithms or SCMs (see also Box 1).

On the data quality side, satellites, as well as station instruments, are plagued by all kinds of measurement errors (Fig. 4, point 11) such as observational noise, systematic biases, and also missing values (notably cloud occlusions or sensor malfunctioning). These may also be causally related to the measured process, constituting a form of selection bias (Fig. 4, point 12).

While in Earth system sciences the data will often attain a continuous range of values (e.g., temperature), variables can also be of a discrete type (Fig. 4, point 13), either due to quantization, or as categorical data. For example, one may be interested in causal drivers of an index representing different weather regimes or a time series of rarely occurring extreme events, which additionally raises the challenge of class imbalance—many 0 and few 1. Causal inference problems with such data require a suitable choice of methods, for example, conditional independence tests adapted to mixed data types. For paleo-climate data, the assumption of a time order is challenged since the measurement time points typically are given only with uncertainty (Fig. 4, point 14).

### Computational and statistical challenges

From a computational and statistical point of view, scalability is a crucial issue, both regarding sample size (Fig. 4, point 15) and high dimensionality (Fig. 4, point 16). While larger sample sizes (long time series) are typically always beneficial for more reliable causal inferences, the computational time of methods may scale unfavorably with sample size (e.g., cubically for some kernel methods^16^). The more variables are taken into account for explaining a potentially spurious relationship, the more credible a causal discovery becomes. However, many variables together with large time lags to account for physical time delays (e.g., to identify atmospheric teleconnections), lead to high dimensionality which may strongly affect statistical reliability. This compromises statistical power, that is, the probability to detect a true causal link, and potentially also the control of false positives at a desired significance level^23,24^. Low-statistical power implies that, especially, weak causal effects with low signal-to-noise ratio, which are sometimes of interest, are not well detected. Last, uncertainty estimation (Fig. 4, point 17) that also takes into account potentially available data uncertainties (measurement value as well as dating uncertainties, see points 11 and 14), poses a major challenge for causal inference methods.

Most of the challenges discussed in this section are the same for correlation or regression methods which are, in addition, ambiguous to interpret and often lead to incorrect conclusions as shown in the examples section. We therefore emphasize that there is no strong reason to avoid adoption and exploration of modern causal inference techniques. Each of the methods summarized in the method overview section addresses one or several of these challenges. In Table 1 we list key strengths and suggest future research directions further discussed in the next section.

**Table 1:** List of methods, key strengths, and further research directions addressing current limitations

Method	Key strengths	Further research directions
Granger causality and nonparametric extensions^9,37,99^	Significance assessment; nonparametric versions	Dealing with contemporaneous effects and feedback cycles; high-dimensionality; deterministic dependencies; synergistic effects; time scales; unobserved variables
Nonlinear state-space methods^10,11^	State-dependent nonlinear systems; contemporaneous effects	Significance assessment; high-dimensionality; highly synchronous dynamics; high stochasticity; time scales; unobserved variables
Conditional independence-based algorithms^12^	High-dimensionality; unobserved variables; nonparametric tests	Significance assessment; deterministic effects; synergistic effects; time scales; contemporaneous feedback cycles
PCMCI^23,24^	High-dimensionality; time delays; strong autocorrelation; nonparametric tests	Unobserved variables; deterministic effects; synergistic effects; time scales; contemporaneous feedback cycles
Information-theoretic algorithms^23,24,51^	High-dimensionality; nonparametric; time delays; information-theoretic interpretation	Significance assessment; unobserved variables; deterministic effects; synergistic effects; time scales; contemporaneous feedback cycles; efficient entropy estimation
Structural causal models^13,38^	Contemporaneous effects; nonparametric versions	High-dimensionality; synergistic effects; time scales; unobserved variables; time delays
Invariance-based methods^4,13,57,58,60,61^	Utilizes heterogeneity in space and time	Causality in stationary regimes; same as for SCMs
Bayesian score-based approaches^48^	Bayesian uncertainty assessment; inclusion of expert knowledge	High-dimensionality; nonlinearity; deterministic effects; synergistic effects; time scales; contemporaneous feedback cycles; unobserved variables; combine with cond. independence-based methods^100^

This table is intended to be a rough method guide. A detailed overview is beyond the scope of this Perspective and hardly possible because comparison studies are currently largely lacking. Spurring research to overcome this lack is a goal of this Perspective and the accompanying platform causeme.net. The terms used in this table are explained in the challenges section and illustrated in Fig. 4

Finally, a crucial challenge when interpreting the output of causal inference methods is that causal conclusions are based on the assumptions underlying the different methods^12,13,24^. These assumptions should, but often cannot, be tested and it is important to make them transparent and discuss how different assumptions would alter conclusions for a particular application.

## Way forward

### Avenues of further methodological research

The preceding Earth system sciences challenges (Fig. 4) are rather generic for complex dynamical systems and apply to many other fields. The challenges point to a way forward to advance causal inference methods for such systems. In the short term, our example applications demonstrate that the existing methods already address some of the mentioned challenges. For example, PCMCI was developed to address high-dimensional time-lagged linear and nonlinear causal discovery and takes into account autocorrelation^23,24^ and CCM^11^ was specifically built to account for nonlinear state-dependent relationships. The largest potential for short-term methodological advancements lies in combining different conceptual approaches in order to address multiple challenges.

First, to give some examples, such as those listed in Table 1, causal network learning algorithms that deal well with high-dimensional data are limited by their inability to identify causal directionality among Markov equivalence classes^12^. This shortcoming can be alleviated by combining causal network learning algorithms with the SCM framework and making additional assumptions on (independence of) mechanisms^4,13,57,83^ that permits to identify causal directions in these cases. Secondly, novel methods can incorporate ideas from theory on causal discovery in the presence of unobserved variables and selection bias^12,47^, time-sub-sampling^84,85^, time-aggregation and cyclic feedbacks^86^, and measurement error^87^. Thirdly, filtering methods as preprocessing steps, e.g., based on wavelets^88^, can help to disentangle causal relations on different time scales, in the simplest example by filtering out a confounder like the seasonal cycle.

In the mid-term, it is worth exploring methods that have not been applied to Earth system data, but whose theoretical properties may render them suitable for the challenges at hand. For example, further methods that are based on the principle of independent mechanisms^4,13,57,83^ such as prediction invariance^13,58,59,61^ or causal discovery from non-stationary data^60^ can potentially make use of the ubiquitously present nonstationarity and external perturbations in Earth system data to infer causal structure. While the black-box character of most machine learning algorithms and deep learning in particular does not lend itself directly to causal discovery, such tools can nevertheless be useful in many aspects of causal discovery. For example, Chalupka et al.^89^ use neural networks to reconstruct causal features from gridded time series datasets. Also conditional independence tests can be based on deep learning^90^ and causal inference can be phrased as a classification problem^91^. And the other way around: causal knowledge, as argued by Pearl, should be incorporated into machine learning to yield more robust predictions and classifications, for example, in such unresolved problems as extrapolation and domain adaptation^14^.

### Validation and a benchmark platform

Method development and comparison require benchmark datasets with known causal ground truth for validation. Ideally, such ground truth comes from expert knowledge on real data or real experiments that can also be used for falsification of causal relationships predicted from observational causal inference methods. Unfortunately, in Earth system sciences such datasets currently exist only for expert-labeled causal relations among few variables (e.g., some bivariate examples in ref. ^92^). To some extent, out-of-sample predictions can provide partial validation, but the main alternative in Earth system sciences is experiments from physical simulation models. Such experiments, however, are computationally expensive and carry the challenge how these have to be designed. A more tractable approach is to generate synthetic data with simple model systems that mimic properties and challenges of geoscientific data, but where the underlying ground truth is known. These can then be used to study the performance of causal inference methods for different challenges in realistic finite sample situations. From a practitioner’s perspective, it is important to find out which method is best suited for a particular task with particular challenges and for a particular set of assumptions. Synthetic data, adapted to the problem at hand, can be used to choose the right method including method parameters. As a first step to close the gap between method users and developers, we accompany this Perspective by a causality benchmark platform (causeme.net) with synthetic models mimicking real data challenges on which causal inference methods can be compared. Next to method comparison, the platform also calls for submissions of real and modeled data sets where the causal structure is known with high confidence. Insights from such benchmark studies are relevant also for many other fields.

### Combining observational causal inference and physical modeling

In the long term, we envision that the two main approaches to understand the Earth system (observational data analysis and Earth system modeling) should become more and more integrated. On the one hand, the generic problem of model evaluation has outlined ways on how causal inference methods can be used to identify weaknesses of physical models and guide model improvement. Furthermore, the currently often heuristic parametrization schemes in physical models can be guided by causal analyses of the respective variables, similar to the proposal to utilize machine learning to systematically replace parametrization schemes^19,93^. Causal discovery can also help to design computationally expensive physical model experiments more efficiently: causal relationships estimated from climate model control runs^79^ (long model runs with fixed pre-industrial conditions) can provide guidance on where numerical experiments are useful and where causal effects are not to be expected.

On the other hand, physical constraints, either from theoretical knowledge or from experimental (modeling) results, can be used to regularize causal inference methods, for example, by defining variables, restricting functional classes, identifying expected noise distributions, time lags and time aggregation, or general data preprocessing. Even more integrated, novel causal inference methods can make combined use of observational as well as experimental data^94,95^ which has already led to fruitful insights in genetics. In Earth system sciences, also information from real experiments on subsystems can be incorporated, not on a large climatic scale^2^, but for example from ecosystem^96^ and mesocosm experiments^97^ in ecological labs.

### Detecting and attributing climate change

Detection and attribution approaches quantify the evidence for a causal link between external drivers of climate change and long-term changes in climatic variables^2^. The goal is to first detect a change and then attribute this change to the contributions of multiple anthropogenic and natural forcings, and from internal variability^2^. Importantly, the focus lies on the effects of long-term forcings on long-term climatic trends or also changes in, e.g., the frequency of extreme weather events. Such research questions require counterfactual worlds, which can only be constructed with climate models, that are then statistically analyzed. For example, the optimal fingerprinting method^2^ is based on attributing detected long-term responses to fingerprint patterns using multiple linear regression. Hannart et al.^98^ discuss the inclusion of Pearl’s^4^ causal counterfactual theory for a more rigorous foundation of detection and attribution studies.

Nevertheless, observational causal inference methods can help to improve climate models as discussed above and can also directly be used to analyze climate feedbacks in paleo-climate data^44^, which is still challenging due to scarce available data and dating uncertainties (Fig. 4). Furthermore, the recent concept of emergent constraints attempts to identify an observable statistical relationship between a feature of interest and a future climate change signal. For example, climate sensitivity, i.e., the response of global mean temperature to greenhouse gas emissions, can be constrained this way^82^. The underlying premise is, however, that today’s dependencies between the predictors and climate sensitivity represent actual physical processes that also hold under future climate change. Here causal discovery can give more robust insights by identifying causal predictors that are more likely to hold under future climate change scenarios.

## Conclusions

The current state-of-the-art in data analysis of the Earth system is still dominated by correlation and regression methods, despite the fact that these methods often lead to ambiguous and confounded results. Existing causality methods can already yield deeper insights from hypothesis testing to the causal evaluation of physical models—if the particular challenges of Earth system sciences are properly addressed. A major impediment to a much wider adoption of causal inference methods is the lack of a reliable benchmark database. We aim to fill this gap by the accompanying platform causeme.net which also includes links to accessible software packages. Applying and interpreting causal inference methods and integrating these with physical modeling, however, will also require more in-depth training on methods in Earth system sciences. Moreover, data-driven causality analyses need to be designed carefully: They should be guided by expert knowledge of the system (requiring expertise from the relevant field) and interpreted based on the assumptions and limitations of the causality method used (requiring expertise from the causal inference method). Sensibly applied causal inference methods promise to substantially advance the state-of-the-art in understanding complex dynamical systems from data also in many other fields with similar challenges as in Earth system sciences, if domain scientists and method developers closely work together—and join the ‘causal revolution’^14^.

## Data Availability

This Perspective is accompanied by a website hosting a causality benchmark platform. causeme.net runs a fair use data policy by which data are made freely available to the public and the scientific community in the belief that their dissemination will lead to greater understanding and new scientific insights and that global scientific problems require international cooperation. Open access means that data are freely distributed without charge. Data download is unrestricted and requires only a free registration for web security reasons. The platform is intended as a system for causal inference method intercomparison in a consistent data environment.
